# The Effect of a Food Addiction Explanation Model for Weight Control and Obesity on Weight Stigma

**DOI:** 10.3390/nu12020294

**Published:** 2020-01-22

**Authors:** Kerry S. O’Brien, Rebecca M. Puhl, Janet D. Latner, Dermot Lynott, Jessica D. Reid, Zarina Vakhitova, John A. Hunter, Damian Scarf, Ruth Jeanes, Ayoub Bouguettaya, Adrian Carter

**Affiliations:** 1 School of Social Sciences, Faculty of Arts, Monash University, Melbourne 3800, Australia; 2 Department of Human Development and Family Sciences, Rudd Center for Food Policy & Obesity, University of Connecticut, Storrs, CT 06269, USA; 3 Department of Psychology, College of Social Sciences, University of Hawaii, Manoa, HI 96822, USA; 4 Department of Psychology, Faculty of Science and Technology University of Lancaster, Lancaster LA1 4YW, UK; 5 Division of Sciences, Department of Psychology, University of Otago, Dunedin 9016, New Zealand; 6 Curriculum & Pedagogy, Faculty of Education, Monash University, Melbourne 3800, Australia; 7 School of Psychology, Faculty of Medicine, Nursing, and Health Sciences, Monash University, Melbourne 3800, Australia

**Keywords:** stigma, obesity, food addiction, weight bias, weight stigma, obesity prejudice reduction

## Abstract

There is increasing scientific and public support for the notion that some foods may be addictive, and that poor weight control and obesity may, for some people, stem from having a food addiction. However, it remains unclear how a food addiction model (FAM) explanation for obesity and weight control will affect weight stigma. In two experiments (*N* = 530 and *N* = 690), we tested the effect of a food addiction explanation for obesity and weight control on weight stigma. In Experiment 1, participants who received a FAM explanation for weight control and obesity reported lower weight stigma scores (e.g., less dislike of ‘fat people’, and lower personal willpower blame) than those receiving an explanation emphasizing diet and exercise (*F*_(4,525)_ = 7.675, *p* = 0.006; and *F*_(4,525)_ = 5.393, *p* = 0.021, respectively). In Experiment 2, there was a significant group difference for the dislike of ‘fat people’ stigma measure (*F*_(5,684)_ = 5.157, *p* = 0.006), but not for personal willpower weight stigma (*F*_(5,684)_ = 0.217, *p* = 0.81). Participants receiving the diet and exercise explanation had greater dislike of ‘fat people’ than those in the FAM explanation and control group (*p* values < 0.05), with no difference between the FAM and control groups (*p* > 0.05). The FAM explanation for weight control and obesity did not increase weight stigma and resulted in lower stigma than the diet and exercise explanation that attributes obesity to personal control. The results highlight the importance of health messaging about the causes of obesity and the need for communications that do not exacerbate weight stigma.

## 1. Introduction

Research on the extent, nature, and impact of weight stigma (also termed weight bias, obesity stigma) suggests that weight stigma has increased over time in adults [1] and children [2] and is associated with a host of negative social and health outcomes [3]. For example, research shows that women perceived to be overweight or obese encounter discrimination in education, health, and employment settings [4,5,6]. Similarly, experiences of weight stigma are associated with poorer psychological and physical outcomes arising from stigma-related stress, including increased depression and anxiety [7], emotional and stress-related eating [8], and avoidance of health care settings [9]. As such, there is a need for research that seeks to understand factors that contribute to and reinforce weight stigma. 

Attribution theories, and specifically, the attribution-value model [10] suggests that antipathy toward a specific group or target is maintained by beliefs about the controllability of specific group behaviours. In the case of overweight and obesity, studies show that weight stigma is increased by attributions about controllability of weight and obesity [11,12]. That is, because people are exposed to public health and media messages that weight is under personal control, people conclude that obesity must be due to an individual’s personal failures, which in turn leads to greater weight stigma [13,14]. Dominant public health messages on the cause of overweight and obesity remain focused, if simplistically, on individual control of diet and physical activity [15]. This individualistic public health narrative is increasingly criticised [16], as it ignores research on the myriad of uncontrollable factors contributing to weight control and obesity, such as neurophysiology, environment, and the interplay with genetics/epigenetics. Experimental evidence suggests that changing people’s attributions about the causes of obesity away from individual blame, and to more biologically and environmentally pre-determined factors, can help to reduce weight stigma [11]. Accordingly, correcting public misattributions about weight has the potential not only to improve knowledge about the complex causes of obesity, but also the potential to reduce weight stigma and discrimination [17].

Recent research posits that some people may have a neurobiological addiction to certain foods, particularly ultra-processed hyper-palatable foods. This addiction may, in part, contribute to people’s food choices and consumption behaviour, and in turn obesity rates [18,19]. Termed *food addiction*, neuropsychological and behavioural research on the addictive properties of food identifies considerable overlap in the food and drug reward and addiction-related centres and pathways of the brain; as a result, food can be as rewarding and addictive as other addictive substances such as drugs, which share overlapping brain reward pathways [20,21]. Large-scale studies suggest that a significant proportion (15%) of the general population [22], and a greater proportion of those with obesity (up to 30%) meet criteria for a diagnosis of food addiction [23]. Furthermore, between 28% and 52% of the general population perceive themselves to be addicted to food [24].

The food addiction model (FAM) for weight control and obesity raises questions about whether a FAM explanation could be helpful or harmful in efforts to reduce weight stigma. While it could be argued that a FAM explanation might increase weight stigma as a result of labelling individuals with obesity as having an addiction, it is possible that a FAM explanation could instead reduce stigma towards people perceived to have obesity by reducing attributions of individual controllability of weight [25]. Research examining these questions is scarce [26]. Some research suggests that the addition of the addiction label to obesity is associated with increased vulnerability to stigmatization [27], and experimental work suggests that the FAM explanation may increase stigma associated with obesity [28]. In contrast, experimental research by Latner and colleagues [25] found that a FAM explanation for weight control and obesity resulted in less stigma and less blame for targets at both lower and higher body weights. These mixed findings indicate the need for additional research to establish whether a FAM explanation increases or decreases weight stigma relative to current public health messaging that suggests diet and exercise as the primary drivers of weight and obesity [29,30].

The present study aimed to examine whether a food addiction explanation for weight control would exacerbate or ameliorate weight stigma relative to the dominant public health messaging emphasizing personal control of diet and exercise. We conducted two experiments to assess the impact of a food addiction explanation for obesity on expressions of weight stigma. In line with previous research with the attribution value model [10] for weight stigma, we predicted that the FAM explanation would result in less weight stigma towards people perceived to be obese or “fat”.

## 2. Methods (Experiment 1 and 2)

### 2.1. Participants

Table 1 details the demographic characteristics of participants in Experiment 1 and 2. For Experiment 1, a sample of *N* = 652 university (college) students was invited to participate in the experiment in return for course credit. Most students (86%; *N* = 561) agreed to participate, with *N* = 530 (94%) of those agreeing to participate providing data on the outcome variables. For Experiment 2, university (college) students (*N* = 717) were invited to participate in the experiment in return for course credit. Most (96%; *N* = 696) agreed to participate, with *N* = 690 providing data on the outcome variables.

### 2.2. Design (Experiments 1 and 2)

Experiment 1 used a between-subjects experimental design to test the effect of a food addiction explanation (*N* = 263) for weight control and obesity vs. the diet and exercise explanation (*N* = 267) on weight stigma (prejudice towards “fat” people). The host university’s Qualtrics research platform randomisation function with a 1:1 ratio was used for randomisation to conditions. Participants received either a simulated news article from The Guardian on the food addiction explanation for weight control and obesity (food addiction condition) or an identically formatted news article positing the dominant public health message that weight control and obesity are due to poor dieting and/or exercise behaviour. Single-item post-manipulation measures were taken for all variables. 

Experiment 2 (*N* = 690) was identical to Experiment 1, but introduced a control group that received no newspaper article. The randomisation ratio was set at 2:1:1 with the control condition (*N* = 346) having two participants for every one participant allocated to the food addiction (*N* = 167) and diet and exercise conditions (*N* = 175).

### 2.3. Manipulation (Food Addiction vs. Diet and Exercise News Articles, vs. Control/No News Article)

Two newspaper articles were constructed for the experiments. The two articles appeared authentic and were structurally identical, using The Guardian newspaper format, with identical author, date/time of publication, word length, and text/photo placement. Both articles contained identical text reporting on research from The Lancet suggesting that mortality from obesity-related diseases was high and, for the first time, greater than mortality from starvation. However, the articles differed considerably in the text regarding explanations for weight control and obesity. 

The *food addiction* article described the concept of food addiction and explained how foods can be addictive through the involvement of the pleasure/reward centres of the brain, and the release of dopamine when eating some foods, which in turn leads to cravings and a vicious cycle of addiction. The article named the originator of the term food addiction, and stated that approximately 20% of the population may have a food addiction, particularly to highly processed or convenience foods. The article also suggested that food addiction was a key factor in weight control and overweight and obesity. 

The *diet and exercise* news article made no reference to food addictions or cravings, but instead focused on people’s lack of physical exercise, sedentary lifestyles, and overconsumption of unhealthy foods. The article stated that these personal behaviours were the cause of obesity. The article cited research from experts stating that more self-control was needed when choosing what foods to eat, and it concluded by stating that diet and exercise programs are our best chance at reducing the obesity epidemic and that people need to get moving more. 

### 2.4. Measures

Along with demographic characteristics including age, sex, height in centimetres, weight in kilograms, and ethnicity, we assessed participant’s weight stigma (i.e., anti-fat prejudice, weight bias). We also included simple measures to assess whether the manipulations affected beliefs about the causes of obesity and weight gain and loss and a food addiction condition manipulation check. All participants received all of the measures summarized below.

#### 2.4.1. Weight Stigma

To measure weight stigma we used the Anti-Fat Attitudes Test (AFAT), a psychometrically sound measure that has been widely used in the field to measure weight bias [31]. The AFAT is a 13-item scale comprised of three subscales assessing dislike of “fat people” which assesses antipathy towards people perceived to be “fat” (Dislike: 7 items, e.g., “I really don’t like fat people much”), fear of becoming fat (Fear of Fat: 3 items, e.g., “I worry about becoming fat”), and belief that excess weight is due to a lack personal willpower (Willpower: 3 items, e.g., “Some people are fat because they have no willpower”). Participants indicate their agreement to items using a scale ranging from 0 = *very strongly disagree* to 9 = *very strongly agree*. The mean of the subscale items is used for analyses. Previous work has identified that the ‘fear of fat’ subscale functions as a measure of personal body image rather than weight stigma toward others per se, with one of the items lacking face validity, so this subscale was not analysed in the present study [32]. Cronbach’s alpha’s for the dislike and willpower subscales were good in the present sample: α = 0.87 and α = 0.79, respectively.

#### 2.4.2. Belief in the Food Addiction Explanation

The food addiction support index (FASI) [24] was used to assess participant beliefs in, and support for, the food addiction explanation for eating, obesity, and weight gain, following exposure to the food addiction vs. diet and exercise news articles (manipulation). Participants responded to the five-item FASI (e.g., “Obesity should be treated as an addiction”), using a five-point scale ranging from 0 = *strongly disagree* to 4 = *strongly agree* with items summed to form a scale total from 0 to 20. Cronbach’s alpha’s in the present experiment was α = 0.83.

#### 2.4.3. Belief in Diet and Exercise for Weight Control

Two items from the dieting beliefs scale [33] that directly capture beliefs about exercise and dieting for the control of weight were used to assess the following exposure to the food addiction vs. diet and exercise news article (manipulation). Specifically, participants were asked to indicate their agreement using a six-point scale ranging from 1 = *not at all descriptive of my beliefs* to 6 = *very descriptive of my beliefs* to the statements “By restricting what one eats, one can lose weight” and “By increasing the amount that one exercises, one can lose weight”. The two items were combined to form a score ranging from 1 to 12, with higher scores indicating greater belief that diet and exercise are responsible for weight control and obesity.

### 2.5. Food Addiction Manipulation Check

To assess whether participants attended to the information in the food addition article, we asked a short question assessing recall for a specific piece of information in the food addiction article. Specifically, we asked participants to identify via a multi-choice recognition response (four answer options) “Who first introduced the term Food Addiction?” Participant responses were coded as either incorrect = 0 or correct = 1.

### 2.6. Procedure

Upon entering the experiment via a web-link to the host university’s Qualtrics research platform, participants in both experiments were provided with the title and description of the study, and then were asked to provide consent to participate. To limit bias in sampling and responding, the study used deception in the advertising and description of the experiment. Participants were told that the experiment was interested in how cognitive information processing styles affect public opinion on a range of political, health, and social issues and that researchers were interested in how people deal with being saturated by the wide range of media messages they receive via TV, computer, and mobile devices.

Participants first answered demographic questions before being presented with one of the two news articles (FAM condition vs. diet/exercise condition) or no article for those in the control group for Experiment 2. To enhance the authenticity of the experiment guise, a large set of distractor questions taken from measures assessing experiential and analytical thinking styles [34] were interspersed in the outcome and manipulation measures. These measures asked participants whether they enjoy the process of thinking deeply about issues, and they have been used successfully elsewhere [35]. Finally, participants were presented with the outcome and manipulation measures. The experiment took approximately 22 min on average to complete. Ethical approval for the study was sought and provided by the host university’s Human Research Ethics Committee (Project ID: 8912).

### 2.7. Analysis

Chi-squared (*X*^2^) and t-tests were used to assess whether randomisation resulted in balanced groups based on gender, age, and body mass index (BMI). Because weight stigma scores were not normally distributed in either experiment, we adopted a two-step transformation to normalise the data [36]. Subsequent normality checks showed the data to have no issues with skewness or kurtosis. ANCOVAs accounting for age, sex, and BMI as covariates were used to test for significant mean group differences on the dislike and willpower weight stigma measures. A Chi-squared test assessed accurate recognition of the food addiction originator in a probe question (manipulation check). ANOVA was also used to examine whether there were any differences on the manipulation measures (i.e., FASI, dieting/exercise beliefs, and food addiction article attention; i.e., who was the originator of food addiction term). We report adjusted means (M) and standard deviations (SD) along with *F* and *p*-values (significance was set at 0.05) for all primary outcomes. 

## 3. Results (Experiments 1 and 2)

### 3.1. Preliminary Analysis Experiment 1

Preliminary analyses (*X*^2^ and *t*-tests) assessing whether randomisation resulted in balanced groups on demographic characteristics showed there were no significant differences between groups for sex, age, or BMI scores (all *p* values > 0.27). A higher proportion of participants in the food addiction condition (61%) correctly recalled the name of the originator of the term food addiction than did those in the diet and exercise condition (39%, *X*^2^ = 23.663, *p* < 0.0005).

### 3.2. Experiment 1 Food Addiction vs. Diet and Exercise

ANCOVA found significant group difference for the ‘dislike’ weight stigma measure: *F*_(4,525)_ = 7.675, *p* = 0.006. Participants exposed to the FAM explanation had significantly lower dislike of “fat people” (M = 1.82, SD = 1.46) scores than did the participants in the diet and exercise condition (M = 2.13, SD = 1.70). Similarly, participants in the FAM group endorsed significantly lower willpower stigma scores (M = 4.29, SD = 205) than participants in the diet and exercise explanation group (M = 4.68, SD = 1.97; *F*_(4,525)_ = 5.393, *p* = 0.021), indicating that those in the FAM condition were less likely to attribute excess weight to a lack of personal willpower.

We examined whether there were differences between groups in the posited beliefs about the causes of weight control and obesity (i.e., FASI, dieting/exercise beliefs). There were statistically significant group difference on FASI scores. Participants receiving the FAM explanation for obesity and weight control had greater belief in the FAM explanation for obesity and weight control (FASI) (M = 14.57, SD = 3.78) than participants receiving the traditional diet and exercise explanation (M = 13.66, SD = 3.80; *F*_(4,525)_ = 8.823, *p* = 0.003). There was not a statistically significant group difference for the dieting and exercise beliefs measure (FAM, M = 8.60, SD = 2.04; diet and exercise M = 8.50, SD = 2.03; *F*_(4,525)_ = 0.100, *p* = 0.75). 

### 3.3. Preliminary Analysis Experiment 2

Preliminary analyses (*X*^2^ and *t*-tests) assessing whether randomisation resulted in balanced groups on demographic characteristics showed there were no significant differences between groups for sex, age, or BMI scores (all *p* values > 0.46). A higher proportion of participants in the food addiction condition (58%) correctly recalled the name of the originator of the term food addiction than did those in the diet and exercise condition (42%; *X*^2^ = 15.047, *p* < 0.0005).

### 3.4. Experiment 2 Food Addiction vs. Diet and Exercise vs. Control/No News Article

ANCOVA found significant group difference for the dislike of ‘fat people’ weight stigma scores (*F*_(5,684)_ = 5.157, *p* = 0.006). Post-hoc tests showed that participants exposed to FAM explanation had significantly lower dislike of ‘fat people’ (M = 1.85, SD = 1.57) than participants in the diet and exercise condition (M = 2.23, SD = 1.76, *p* < 0.001). Participants in the diet and exercise condition also endorsed higher weight stigma scores than those in the control condition (M = 1.70, SD = 1.54). There was no significant difference in dislike of ‘fat people’ scores between the FAM condition and the control group (*p* = 0.56). There was also no significant group difference for willpower weight stigma scores (*F_(_*_5,684)_ = 0.217, *p* = 0.81), with the FAM, diet and exercise, and control groups having similar mean scores (M = 4.71, SD = 2.05, and M = 4.81, SD = 1.86, M = 4.62, SD = 2.16, respectively).

We examined whether there were differences between groups in the posited beliefs about the causes of weight control and obesity (i.e., dieting/exercise beliefs, FASI). There was no significant group difference for the dieting/exercise beliefs measure (*F*_(5,682)_ = 2.055, *p* = 0.13; diet and exercise condition M = 4.62, SD = 0.95, control condition M = 4.38, SD = 1.06, FAM group M = 4.43, SD = 1.03). We found no statistically significant group difference in FASI scores (*F*_(5,680)_ = 1.764, *p* = 0.17; control M = 14.54, SD = 4.17, FAM M = 15.01, SD = 3.29, diet and exercise M = 13.99, SD = 3.90). 

## 4. Discussion

We examined whether the FAM explanation for weight control and obesity versus the traditional public health messaging around control of diet and exercise would affect weight stigma. Relative to the dominant public health narrative that obesity stems from lack of control over diet and exercise, the FAM explanation resulted in lower weight stigma. In Experiment 1, both dislike of ‘fat people’ and perceptions that excess weight is a result of lack of willpower were lower in people presented with the FAM explanation. In a second experiment, we introduced a control group that was not exposed to information about obesity or causal models for obesity. Similar to Experiment 1, participants in Experiment 2 who received the FAM explanation for weight control and obesity displayed less weight stigma (dislike of “fat people”) than participants in the diet and exercise condition. Importantly, there was no difference between the FAM and control groups in levels of weight stigma. Contrary to Experiment 1, in Experiment 2 there was no significant group difference with respect to perceptions that excess weight is caused by a lack of willpower (blame). The results of these two experiments are consistent with work by Latner and colleagues [25] who reported less stigma following exposure to a food addiction explanation. The results do not appear to support the notion that attaching an addiction label to people with obesity would exacerbate weight stigma. Accordingly, a simple interpretation of the results of both experiments is that the dominant public health messaging around the diet and exercise explanation for weight control and obesity exacerbates weight stigma, but the FAM explanation does not. 

We found mixed support for the attribution value model of weight stigma [10]. Analysis of the FASI scores in Experiment 1 suggest that the difference in weight stigma scores between the FAM and diet and exercise conditions was due to changes in participants’ attributions about the causes of obesity. However, we found no significant group difference on FASI scores in Experiment 2. Similarly, there was no group differences in participant beliefs about personal control of diet and exercise as a primary cause for obesity and weight control in Experiment 1 or 2. This finding is unexpected, as it was reasonable to predict a decrease in attributions of dieting and exercise as a causal explanation among participants who received the FAM explanation. It is possible that the FAM explanation resulted in less stigma because of a greater understanding of, and/or empathy for, those facing the challenges of weight management when one is addicted to food. As we did not assess constructs related to empathy, it will be important for future work to assess the relationship between perceptions of food addiction and levels of empathy towards people with obesity. It is apparent that those in the FAM condition did not dismiss the notion of diet and exercise as contributors to weight control and obesity. Participants may already have firmly established beliefs about personal control of weight given pervasive societal messages emphasizing this message. However, this possible pre-existing belief did not interfere with participants’ abilities to receive and incorporate a different message about the contributors to obesity emphasized in the FAM perspective.

The perception that weight is determined by the individual’s personal control of choices regarding dieting and exercise behaviours is widespread and accepted in society [37]. Belief in this dominant public health model for overweight and obesity is thought to be linked to weight stigma because it infers and/or attributes overweight and obesity to personal responsibility, lack of discipline, and laziness. The FAM explanation for obesity and weight control is garnering attention in several research fields [19,38,39] and is gaining traction in popular culture [40]. Indeed, studies suggest that around 15% of people meet criteria for a food addiction and anywhere from 28–52% of people believe they may be addicted to a food [22,23,24]. The present findings support suggestions that popular societal messages of blame and personal responsibility for weight may be partly responsible for the prevalence and rise of weight bias [1]. In contrast, an alternative explanation for obesity, the FAM explanation, may have a positive effect of reducing weight stigma. As our studies did not assess attitudes over time, it will be important for future work to examine whether changes in participants’ causal attributions for obesity can be maintained following brief interventions or information about alternative contributors to obesity. It is likely that repeated exposure to FAM information and messages would be needed to sustain shifts in people’s perspectives over time, especially in the face of continued and prominent societal messages emphasizing personal behaviours as the primary cause of obesity. Nevertheless, findings from both of our studies suggest that it is possible to shift weight stigma attitudes with a brief intervention emphasizing a FAM narrative for obesity.

Several limitations of this research should be noted. First, study participants were mostly young and female, with a majority who did not have overweight or obesity. Future studies should examine the FAM explanation in a more representative population sample, including those with diverse body sizes. Baseline data on weight stigma variables were not collected, which could otherwise have been included as potential covariates in the analyses. However, the decision not to collect baseline data on these variables was balanced against the importance of maintaining the study’s deception, to avoid tipping off participants to the nature of the study and cuing them in the direction of socially desirable responding. Still, future studies could employ within-participant designs to examine potential changes in weight stigma across subjects upon exposure to the FAM model of weight. In addition, future research should explore the longer-term outcomes of public health messages, particularly those delivered in real-world settings.

## 5. Conclusions

The present study found lower weight stigma after exposure to a food addiction model of weight and greater weight stigma after exposure to a diet and exercise model of weight. While several studies have attempted to reduce weight stigma, with varying success, far more work is needed to address this prevalent and harmful societal problem. The improvement found in weight stigma following an addiction explanation, and the worsening of weight stigma following a diet and exercise explanation, has implications for public health messages about body weight and obesity. The results support the growing popularity of the food addiction model for eating and associated body weight and obesity. Increasing public understanding of the role of a food addiction explanation for eating behaviour and weight may help to alleviate weight stigma, including, potentially, internalised weight bias/self-stigma. At the same time, our research suggests that the current dominant public health message that largely attributes weight control and obesity to lack of personal control of, and responsibility for, diet and exercise needs to be changed as it appears to be supporting weight stigma [41]. Future research is needed to explore ways to modify current public health messaging so that it is not exacerbating weight stigma. Such messaging could describe the interplay between biological (e.g., FAM, genes) and/or environmental factors (e.g., food security, access to healthy and affordable foods, exercise-facilitating living and work environments) contributing to weight, as well as the importance of healthy eating and physical activity for all individuals, regardless of body size.

## Figures and Tables

**Table 1:** Participant characteristics for Experiment 1 and Experiment 2.

Parameter	Value
Experiment 1 (*N* = 530)	
Age (years)	M = 19.7, SD = 1.8 (range: 18–35)
Gender (female/male)	73.8% (*n* = 391)/26.2% (*n* = 139)
Body Mass Index (BMI)	M = 22.5, SD = 4.1
Percentage underweight (BMI ≤ 18.5 kg/m^2^)	10.9% (*n* = 57)
Percentage normal weight (BMI = 18.5 < 25.0)	69.4% (*n* = 367)
Percentage overweight or obese (BMI > 25+)	20% (*n* = 106)
Experiment 2 (*N* = 690)	
Age (years)	M = 19.7, SD = 2.51 (range: 18–52)
Gender (female/male)	72.2% (*n* = 498)/27.7% (*n* = 191)
Body Mass Index (BMI)	M = 22.4, SD = 4.1
Percentage underweight (BMI ≤ 18.5 kg/m^2^)	11.1% (*n* = 77)
Percentage normal weight (BMI = 18.5 < 25.0)	68.0% (*n* = 469)
Percentage overweight or obese (BMI > 25+)	20.9% (*n* = 144)

A priori sample size calculations indicated a required minimum sample of *N* = 142 for Experiment 1 and *N* = 216 for Experiment 2 (72 per group) to detect a small to moderate effect size (*d* = 0.30) between experimental groups with desired power at 0.80 and α set at 0.05 (two-sided). The present sample sizes were sufficient for experimental designs and planned analyses.
